# Correction to “Multi‐level metabolic engineering of *Escherichia coli* for high‐titre biosynthesis of 2′‐fucosyllactose and 3‐fucosyllactose”

**DOI:** 10.1111/1751-7915.14451

**Published:** 2024-04-10

Li M, Li C, Luo Y, Hu M, Liu Z, Zhang T. Multi‐level metabolic engineering of *Escherichia coli* for high‐titre biosynthesis of 2′‐fucosyllactose and 3‐fucosyllactose. *Microb Biotechnol*. 2022;15(12):2970–2981. DOI: 10.1111/1751‐7915.14152

In paragraph 3 of the “Results and Discussion” section, the text “with a maximum increase to 2.62 and 0.91 g/L, an increase of 263% and 49% compared to EC04 and EM04 strains (Figure 3C,E).” was incorrect. This should have read: “with a maximum increase to 2.62 and 1.46 g/L, an increase of 263% and 138% compared to EC04 and EM04 strains (Figure 3C,E).”

In paragraph 3 of the “Results and Discussion” section, the text “Deletion of *nudD* (EC10 and EM10 strains) resulted in a 216% and 96% increase in 2′‐FL and 3‐FL titers, reaching 2.28 and 1.20 g/L, respectively.” was incorrect. This should have read: “Deletion of *nudD* (EC15 and EM13 strains) resulted in a 298% and 96% increase in 2′‐FL and 3‐FL titers, reaching 2.88 and 1.20 g/L, respectively.”

In Figure S1 of the “Supporting Information” section, the figure legend “Identification of 2′‐FL and 3‐FL by MS.” was incorrect. This should have read: “Identification of 2′‐FL and 3‐FL by the secondary mass spectrometry.”

Figures 5 and 6 in the “Results and Discussion” section need to be corrected. In updated Figure 5B,D, the titers of 2′‐FL strains containing RBS (BBa_B0034) and the fusion peptide pelB were 7.87 g/L and 8.57 g/L, respectively. The titers of these two strains in the original Figure 5B,D were 8.21 g/L and 9.26 g/L, respectively. In updated Figure 6, the strains obtained 64.62 g/L of 2′‐FL and 40.68 g/L of 3‐FL in fed‐batch fermentation. In the original Figure 6, the two strains obtained 54.82 g/L 2′‐FL and 34.2 g/L 3‐FL in fed‐batch fermentation. The updated data in Figures 5 and 6 are consistent with the results in the main text. The above errors are a confounding of the results of different production batches of strains and replicate experiments. The errors occurred during the final uploading of the figures. These corrections will not have any impact on the main findings of the article.

Below is an updated Figure 5:

**FIGURE 5:**
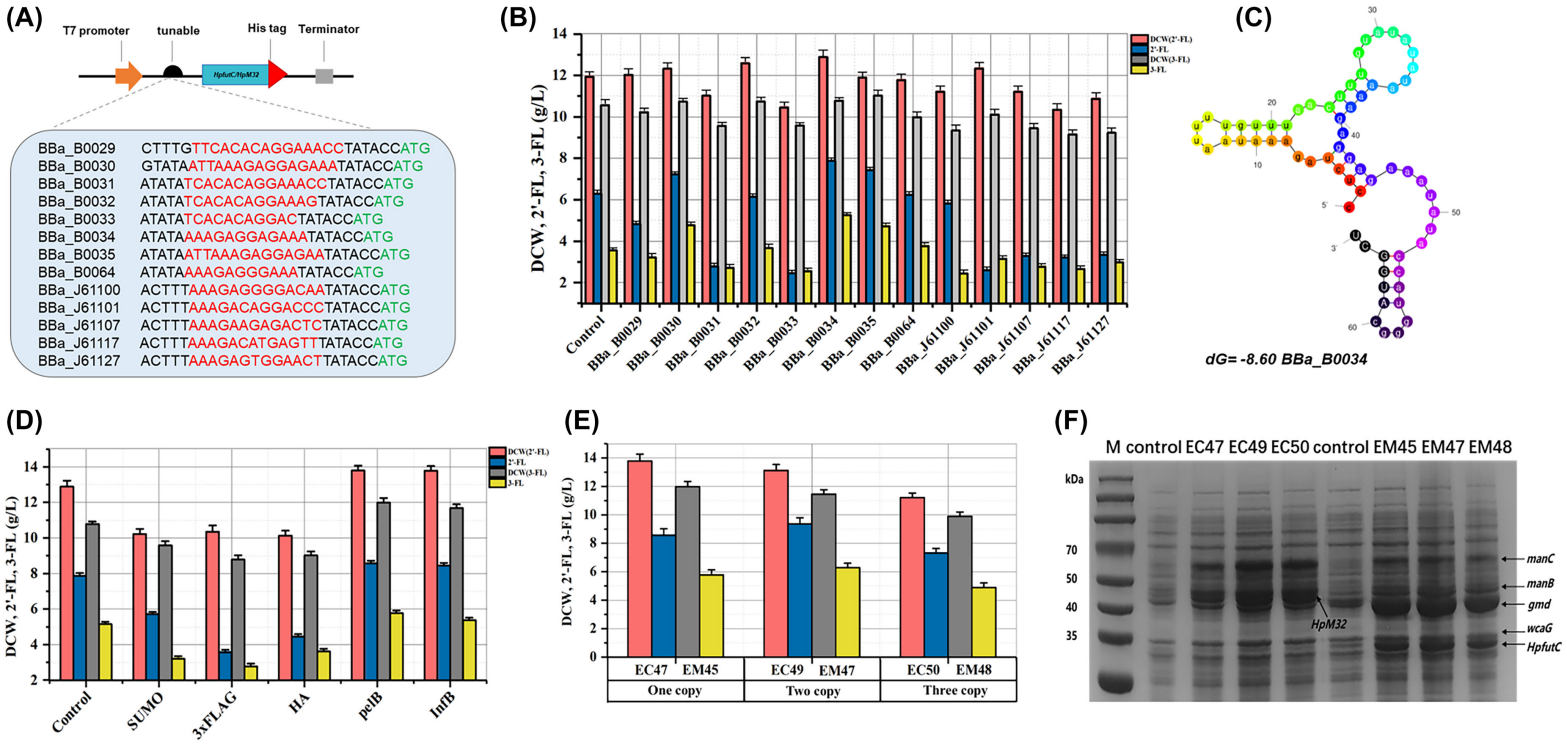
Enhancing the activity of HpfutC and HpM32 by RBS, fusion peptides and multi‐copy gene screening. (A) Schematic diagram of the construction of RBS‐linked HpfutC/HpM32. The selected RBS were from the MIT Standard Biological Parts Registry. The RBS core sequence is coloured in red. The start codon is shown in green. (B) Biomass and 2′‐FL/3‐FL productivity of engineered strains at different intensities of RBS. (C) RNA secondary structure of BBa_B0034 in the 5′ end of mRNA transcripts of BBa_B0034 predicted by the mfold Web Server, purple‐RBS sequence, black‐the start codon. (D) Biomass and 2′‐FL/3‐FL productivity of defective strains under different fusion peptides in batch fermentation. (E) Effect of HpfutC and HpM32 copy numbers on the growth conditions and 2′‐FL/3‐FL productivity of recombinant strains. (F) SDS‐PAGE analysis of expression products in recombinant strains containing different HpfutC and HpM32 copy numbers.

Below is an updated Figure 6:

**FIGURE 6:**
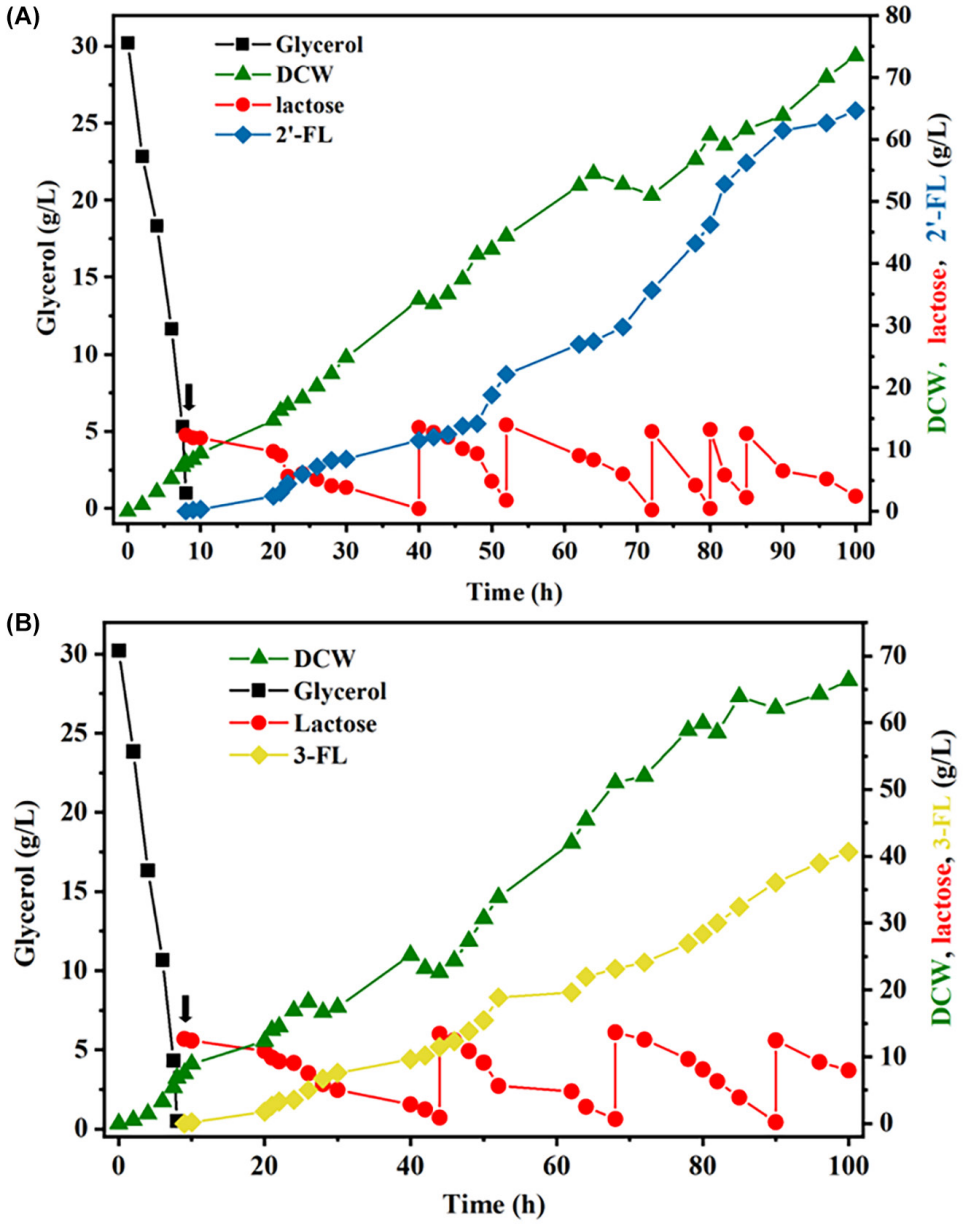
Biosynthesis of FL by fed‐batch cultivation in 3‐L bioreactors. (A) Fed‐batch fermentation of EC49 strain for 2′‐FL production. (B) Fed‐batch fermentation of EM47 strain for 3‐FL production. Thick arrow, IPTG induction and initial addition of lactose. Lactose is intermittently supplemented during the fermentation process.

We apologize for these errors.

